# Exposure–response relationship of olaratumab for survival outcomes and safety when combined with doxorubicin in patients with soft tissue sarcoma

**DOI:** 10.1007/s00280-018-3723-4

**Published:** 2018-11-08

**Authors:** Robin L. Jones, Gary Mo, John R. Baldwin, Patrick M. Peterson, Robert L. Ilaria, Ilaria Conti, Damien M. Cronier, William D. Tap

**Affiliations:** 1 University of Washington and Fred Hutchinson Cancer Research Center, 1100 Fairview Ave N, Seattle, WA 98109 USA; 2 Eli Lilly and Company, Lilly Corporate Center, 46285 Indianapolis, IN USA; 3 Eli Lilly and Company, Lilly Research Centre, Erl Wood Manor Sunninghill Road, Windlesham, Surrey GU20 6PH UK; 4 Memorial Sloan Kettering Cancer Center and Weill Cornell Medical College, 1275 York Ave, New York, NY 10065 USA; 5 Present Address: Royal Marsden Hospital and Institute of Cancer Research, London, UK; 6 Present Address: Celgene, Summit, NJ USA; 7 Present Address: EMD Serono Research & Development Institute, Billerica, MA USA; 8 Present Address: Merck Serono Ltd, Feltham, UK

**Keywords:** Olaratumab, Doxorubicin, Exposure response, Outcome, Soft tissue sarcomas

## Abstract

**Purpose:**

Olaratumab is a recombinant human IgG1 monoclonal antibody against PGDFRα. Olaratumab plus doxorubicin improved survivalversus doxorubicin in an open-label, randomised phase 2 soft tissue sarcoma (STS) trial. We characterised the olaratumab exposure–response relationship for progression-free survival (PFS), overall survival (OS), and safety.

**Methods:**

PFS and OS data from the 133 patients enrolled in the phase 2 study were analysed using time-to-event modelling. The effect of olaratumab on PFS/OS was explored using the trough serum concentration after cycle 1 (C_min1_) and the average concentration throughout treatment (C_avg_). The rate of treatment-emergent adverse events (TEAEs) was compared across olaratumab exposure quartiles.

**Results:**

PFS and OS were described by models with an exponential hazard function and inhibitory E_MAX_ functions to describe the effect of olaratumab, regardless of the PK endpoint. The olaratumab EC50s for PFS (EC_min1_50 = 82.0 µg/mL, EC_avg_50 = 179 µg/mL) and OS (EC_min1_50 = 66.1 µg/mL, EC_avg_50 = 134 µg/mL) corresponded to the median and 25th percentile of C_min1_/C_avg_ in the study, respectively. Maximum predicted improvement in the hazard ratio for OS and PFS was approximately 75% and 60%, respectively. There was no change in the rate of TEAEs with increasing olaratumab serum levels.

**Conclusions:**

PFS/OS benefits occurred without a rate change in TEAEs across quartiles. Maximum benefit in OS was achieved in the upper three quartiles and a potential of early disease progression in the lower quartile of olaratumab serum exposure. These results prompted a loading dose strategy in the ongoing phase 3 STS trial.

## Introduction

Soft tissue sarcomas (STS) are a group of rare tumours of mesenchymal origin, accounting for approximately 1% of all adult cancers [1–3]. For most histological subtypes, the standard management of localised disease consists of complete surgical resection with or without radiation. Despite optimal management, however, high-risk patients will develop recurrent locally advanced inoperable or metastatic disease. The outcome for patients with advanced inoperable/metastatic STS is poor with a median overall survival (OS) in the range of 12–18 months [4–8]. There are few treatment options available, and these have historically included doxorubicin with or without ifosfamide. Over the last few years, a number of other drugs have emerged including gemcitabine/docetaxel, trabectedin, pazopanib, and eribulin [6, 9–12].

Olaratumab is a recombinant human immunoglobulin G1 monoclonal antibody to platelet-derived growth factor receptor alpha (PDGFRα) [13]. A randomised phase 2 trial of doxorubicin with or without olaratumab in patients with advanced STS demonstrated a significantly longer median OS for the combination of doxorubicin and olaratumab compared to doxorubicin alone (26.5 and 14.7 months, respectively, hazard ratio [HR] 0.46, *p* = 0.0003) [14]. The increase in progression-free survival (PFS) was also significant (6.6 months and 4.1 months, respectively, HR 0.67, *p* = 0.0615) and the combination of olaratumab with doxorubicin led to a slight increase in toxicity but remained well-tolerated. Based on this phase 2 STS trial, olaratumab was granted accelerated/conditional approval by a number of regulatory agencies.

A matched case–control analysis [15] performed on the phase 2 PFS and OS survival data stratified by quartiles of olaratumab serum exposure indicated that patients in the lowest quartile may not have received optimal level of clinical benefit [14]. A population pharmacokinetic (PopPK) analysis subsequently performed using PK data combined from four phase 2 studies, including that in STS, indicated that the dose of 15 mg/kg administered on Days 1 and 8 of a 21-day cycle yields olaratumab serum levels likely to achieve full target saturation [16]. In light of these findings, it seems necessary to better define the therapeutic window of olaratumab and determine whether the dose of 15 mg/kg used in the phase 2 study represents the optimal dose to be used in combination with doxorubicin in STS patients. The aim of this study was therefore to characterise the exposure–response relationship of olaratumab in combination with doxorubicin for PFS, OS, and safety for patients with advanced STS.

## Materials and methods

### Clinical trial and data

OS, PFS, and safety data were obtained for the 133 patients enrolled in a randomised, open-label, multicenter, phase 2 trial where the efficacy of olaratumab in combination with doxorubicin was tested in patients with histologically confirmed locally advanced or metastatic STS (NCT01185964) [14]. Patients were randomly assigned in a 1:1 ratio to receive olaratumab (15 mg/kg) intravenously on Day 1 and Day 8 plus doxorubicin (75 mg/m^2^) (*n* = 66) or doxorubicin alone (75 mg/m^2^) on Day 1 of each 21-day cycle for up to eight cycles (*n* = 67). Randomization was stratified according to number of previous lines of treatment (0 versus 1 + lines), histological tumour type (that is, leiomyosarcoma versus synovial sarcoma versus other tumour type), Eastern Cooperative Oncology Group performance status (ECOG PS) (0, 1 versus 2), number of prior lines of treatment (0 versus ≥ 1), and PDGFRα expression (positive versus negative). Throughout the study, patients were assessed for tumour response every 6 weeks according to RECIST 1.1. Patients on the investigational arm without disease progression could continue to receive olaratumab until the development of unacceptable toxicity, noncompliance or withdrawal of consent by the patient, or investigator decision.

The study was conducted in accordance with the Declaration of Helsinki and the International Conference on Harmonisation Guidelines for Good Clinical Practice. The local institutional review boards at each participating study site approved the study protocol, and all patients in the study provided written informed consent to participate.

### Olaratumab PK endpoints

The effect of olaratumab serum exposure on clinical outcome was described using two PK endpoints: the trough concentration at the end of the first cycle of treatment (*C*_min1_), and the average concentration over each patient’s entire treatment (*C*_avg_). These endpoints were selected primarily because they allow the description of olaratumab serum exposure in two different methods: *C*_min1_ describes the intended serum exposure prior to any dose reductions, whereas *C*_avg_ summarises serum exposure retrospectively and captures the impact of dose reductions during the course of treatment. Individual estimates of *C*_min1_ and *C*_avg_ for each patient in the experimental arm were obtained from the population PK model developed using PK data combined from a total of four phase 2 studies, including that in STS patients [16].

### Survival models for OS and PFS

OS and PFS were described by means of parametric time-to-event modeling, where survival was calculated as the inverse of the exponent of the cumulative hazard over time during the study. Both OS and PFS were best described by a time-to-event model where the baseline hazard remains constant throughout the study, so that the time to event (survival duration) is exponentially distributed over time. The effect of olaratumab *C*_min1_ and *C*_avg_ was incorporated into the OS and PFS models as a fractional decrease to the hazard function. Upon establishment of the appropriate survival model, the intrinsic and extrinsic patient factors listed in Table 1 were tested as covariates for their influence on OS and PFS.

**Table 1:** Patient factors assessed in the population pharmacodynamic analysis

Covariate	Type	Parameters tested
ECOG group	Categorical	BASE_HAZ, *E*_MAX_, EC50
Tumour size	Continuous	BASE_HAZ, *E*_MAX_, EC50
Tumour histology	Categorical	BASE_HAZ, *E*_MAX_, EC50
Age group	Categorical	BASE_HAZ, *E*_MAX_, EC50
Gender	Categorical	BASE_HAZ, *E*_MAX_, EC50
Race	Categorical	BASE_HAZ, *E*_MAX_, EC50
Body weight	Categorical	BASE_HAZ, *E*_MAX_, EC50
Prior treatment	Categorical	BASE_HAZ, *E*_MAX_, EC50
Hemoglobin	Continuous	BASE_HAZ, *E*_MAX_, EC50
Albumin	Continuous	BASE_HAZ, *E*_MAX_, EC50

*ECOG* Eastern Cooperative Oncology Group, *BASE_HAZ* baseline hazard, *E*_*MAX*_ maximum response achievable from a dose, *EC50* concentration of a drug that gives half-maximal response

### Exposure–response for safety

The overall incidence of treatment-emergent adverse events (TEAEs) in the investigational arm of the study was stratified by grade (≤ grade 2, > grade 2, ≥ grade 4) and by quartile of olaratumab *C*_min1_ and *C*_avg_ in a tabular format and compared to that in the control arm. The rates of neutropenia and mucositis were also examined in a similar manner.

## Results

### Overall survival model

**Fig. 1:**
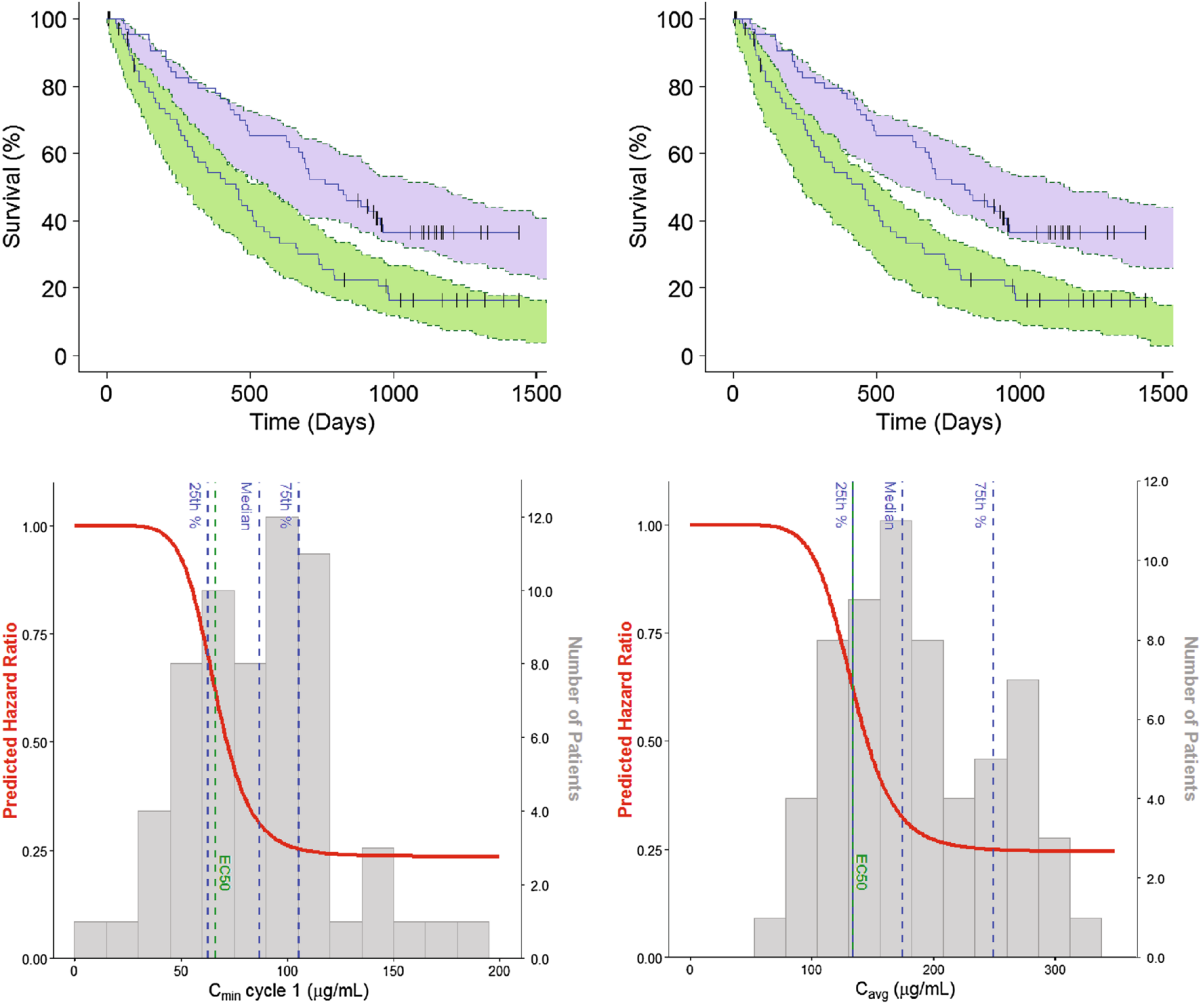
Visual predictive check and model prediction of the overall survival models. **a** Left graph is the VPC of the *C*_min1_-based OS model. Right graph is the VPC of the *C*_avg_-based OS model. The shaded areas indicate the predicted OS in the control (green) and experimental (blue) arm; the solid blue lines describe the corresponding observed OS signals. **b** Overall survival as predicted by *C*_min1_-based model (left panel) and the *C*_avg_-based model (right panel). The solid red lines describe the change in HR as a function of *C*_min1_ and *C*_avg_; the grey histograms describe the distribution of olaratumab *C*_min1_ and *C*_avg_ in the study JGDG experimental arm together with their quartiles (dashed blue lines); the green dashed lines indicate EC_min1_50 and EC_avg_50. C_avg_ = average concentration over patient’s entire treatment. *C*_min1_ = trough serum concentration at the end of Cycle 1. EC_avg_50 = olaratumab C_avg_ yielding a 50% decrease in the baseline hazard. EC_min1_50=olaratumab *C*_min1_ yielding a 50% decrease in the baseline hazard. *HR* hazard ratio, *OS* overall survival, *VPC* visual predictive check

OS in the study was best described by a time-to-event model with an exponential hazard function. In both the *C*_min1_- and *C*_avg_-based models, an inhibitory E_MAX_ drug effect model on the hazard function performed the best to describe the effect of olaratumab. The visual predictive check (VPC) showed good agreement between observed data and model prediction (Fig. 1a). The precision of the parameter estimates was verified by bootstrap analysis, except for the Hill coefficient, which was fixed to allow model stability. All parameter estimates for both OS models are listed in Table 2. *C*_min1_ and *C*_avg_ had a similar predictive role for drug effect, so that the half-maximum effective *C*_min1_ and *C*_avg_ (EC_min1_50 and EC_avg_50) estimates corresponded to the 25th percentile of *C*_min1_ and *C*_avg_ in the study, respectively. The maximum effect (*E*_MAX_) estimates corresponded to a predicted improvement in the hazard ratio of approximately 75% (Fig. 1b). Consistent with the high value for the Hill coefficient, there was no notable change predicted in the HR for patients in the lowest quartile of olaratumab exposure, whereas E_MAX_ was reached within the range of olaratumab serum concentrations achieved in the study. The ECOG PS and the number of prior treatments were found to be significant covariates affecting the baseline hazard for both the *C*_min1_- and *C*_avg_-based models. Patients with an ECOG PS ≥ 1 have a predicted 86.2% increase in the baseline hazard and patients who had received no prior lines of treatment had a 58.3% decrease in the baseline hazard.

**Table 2:** Parameter estimates and bootstrap results for the OS models

Parameters	Population estimate (%SEE)		Bootstrap parameter results (5–95 percentile)	
	C_min1_ model	C_avg_ model	C_min1_ model	C_avg_ model
**Baseline hazard**				
Base_Hazard_	0.00205 (2.18)	0.00203 (2.26)	0.00206 (0.00158–0.00280)	0.00204 (0.00156–0.00277)
**Olaratumab effect**				
E_MAX_	0.765 (8.63)	0.756 (9.07)	0.771 (0.625–0.884)	0.761 (0.607–0.876)
EC_min1_50 (µg/mL)	66.1 (12.1)	–	65.9 (50.9–80.3)	–
EC_avg_50 (µg/mL)	–	134 (6.72)	–	135 (115–163)
Hill	8 (fixed)	8 (fixed)	8 (fixed)	8 (fixed)
**Covariate effects**				
EGRP_Base_	0.862 (42.1)	0.802 (44.0)	0.925 (0.273–1.83)	0.857 (0.220–1.72)
PRVTRT_Base_	− 0.583 (15.9)	− 0.535 (19.1)	− 0.57 − (0.740–0.345)	− 0.528 − (0.706–0.282)

*OS* overall survival, *SEE* standard error of the estimate, *C*_min1_ trough serum concentration at the end of Cycle 1, *C*_avg_ average concentration over patient’s entire treatment, *E*_*MAX*_ maximum response achievable from a dose, *EC*_*min1*_*50* olaratumab C_min1_ yielding a 50% decrease in the baseline hazard, *EC*_*avg*_*50* olaratumab C_avg_ yielding a 50% decrease in the baseline hazard, *EGRP*_*Base*_ covariate effect of ECOG status on the baseline hazard, *PRVTRT*_*Base*_ covariate effect of the number of prior treatment on the baseline hazard

### Progression-free survival model

**Fig. 2:**
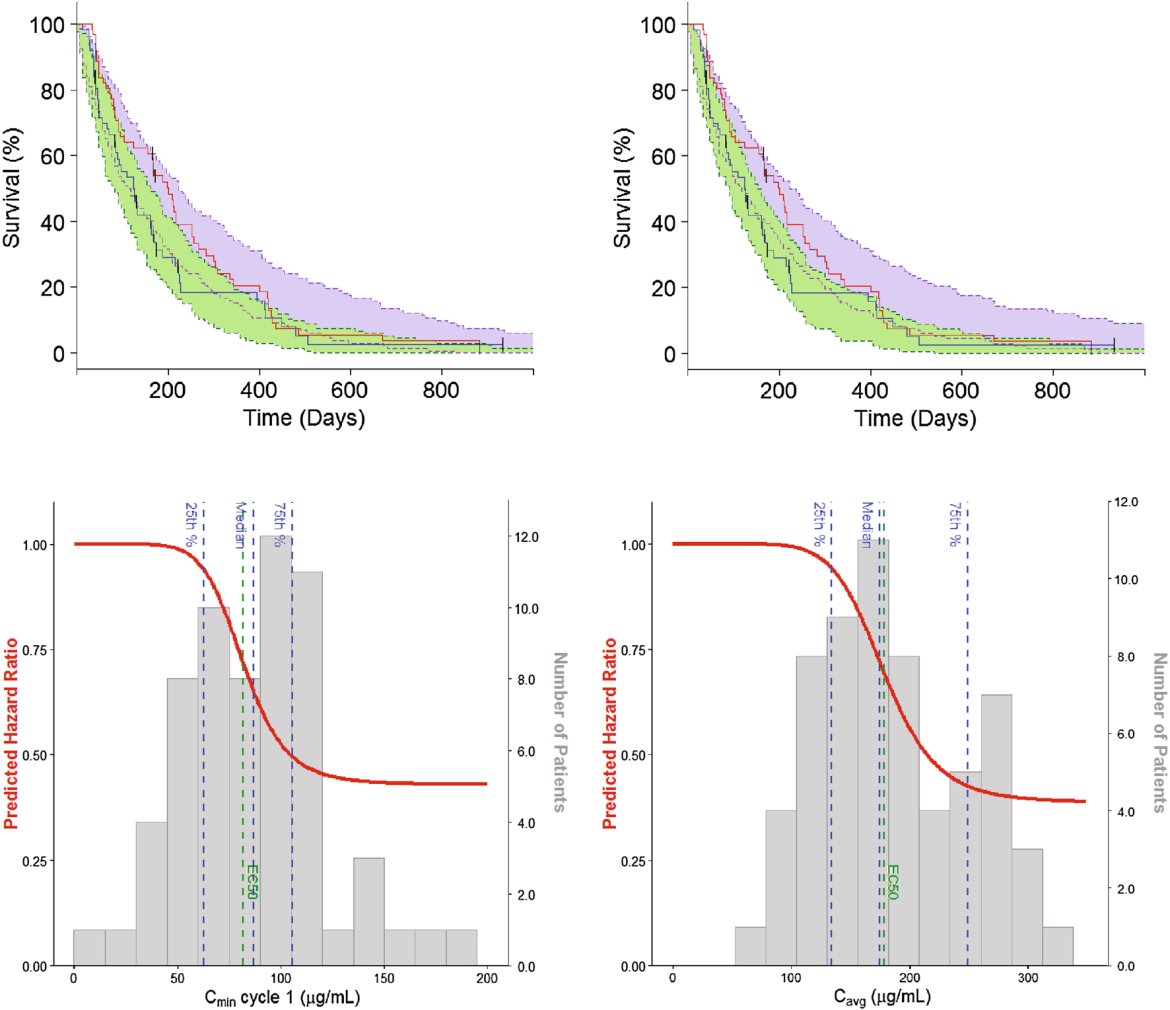
Visual predictive check and model prediction of the progression-free survival models. **a** Left graph is the VPC of the *C*_min1_-based PFS model. Right graph is the VPC of the *C*_avg_-based PFS models. The shaded areas indicate the predicted PFS in the control (green) and experimental (blue) arm; the solid blue lines describe the corresponding observed PFS signals. *C*_avg_ = average concentration over patient’s entire treatment. **b** Progression-free survival as predicted by *C*_min1_-based model (left panel) and the *C*_avg_-based model (right panel). The solid red lines describe the change in HR as a function of *C*_min1_ and *C*_avg_; the grey histograms describe the distribution of olaratumab *C*_min1_ and *C*_avg_ in the study JGDG experimental arm together with their quartiles (dashed blue lines); the green dashed lines indicate EC_min1_50 and EC_avg_50. C_min1_ = trough serum concentration at the end of Cycle 1. EC_avg_50 = olaratumab C_avg_ yielding a 50% decrease in the baseline hazard. *EC*_*min1*_*50* olaratumab *C*_min1_ yielding a 50% decrease in the baseline hazard, *HR* hazard ratio, *PFS* progression-free survival, *VPC* visual predictive check

PFS was best described by a model with a structure similar to that used for OS. Parameter estimates are listed in Table 3 and the model VPC is shown in Fig. 2a. Again, *C*_min1_ and *C*_avg_ had a similar predictive role for drug effect. The EC_min1_50 and EC_avg_50 estimates corresponded to the median of *C*_min1_ and *C*_avg_ in the study, rather than their 25th percentile (Fig. 2b). The maximum predicted effect of olaratumab on PFS was also lower compared to OS, with E_MAX_ estimates of approximately 0.60. None of the covariates explored were found to have a significant effect on PFS in our analysis.

**Table 3:** Parameter estimates and bootstrap results for the PFS models

Parameters	Population estimate (%SEE)		Bootstrap parameter results (5–95 Percentile)	
	C_min1_ model	C_avg_ model	C_min1_ model	C_avg_ model
Baseline hazard				
Base_Hazard_	0.00604 (2–52)	0.00616 (2.63)	0.00607 (0.00473–0.00786)	0.00619 (0.00476–0.00805)
Olaratumab effect				
E_MAX_	0.571 (15–4)	0.614 (14.5)	0.567 (0.363–0.723)	0.617 (0.435–0.770)
EC_min1_50 (µg/mL)	82.0 (6–15)	–	82.3 (69.3–95.4)	
EC_avg_50 (µg/mL)	–	179 (9.33)		182 (149–241)
Hill	8 (fixed)	8 (fixed)	8 (fixed)	8 (fixed)

*PFS* progression-free survival, *SEE* standard error of the estimate, *C*_*min1*_ trough serum concentration at the end of Cycle 1, *C*_*avg*_ average concentration over patient’s entire treatment, *E*_*MAX*_maximum response achievable from a dose, *EC*_*min1*_*50* olaratumab C_min1_ yielding a 50% decrease in the baseline hazard, *EC*_*avg*_*50* olaratumab C_avg_ yielding a 50% decrease in the baseline hazard

### Exposure–response for safety

The incidence of TEAEs in the phase 2 trial stratified by grade and by olaratumab C_min1_ and C_avg_ quartiles are listed in Table 4. The addition of olaratumab to doxorubicin led to a moderate increase in the rate of TEAEs compared to doxorubicin alone, consistent with previous reports. However, when examined across quartiles of olaratumab serum exposure, there was no change in the rate of TEAEs with increasing olaratumab serum concentrations in the investigational arm, regardless of the PK endpoint considered.

**Table 4:** Treatment-related adverse events stratified by olaratumab concentration

	Olaratumab (C_min1_)						Olaratumab (C_avg_)					
	Dox	Overall	Q1	Q2	Q3	Q4	Dox	Overall	Q1	Q2	Q3	Q4
	*n* = 65	*n* = 62	*n* = 15	*n* = 16	*n* = 15	*n* = 16	*n* = 65	*n* = 62	*n* = 15	*n* = 16	*n* = 15	*n* = 16
C_min1_ range (µg/mL)	–	12.3–188.1	12.3–<62.8	62.8–<86.9	86.9–<105.6	105.6–188.1	–	56.0–347.3	56.0–<134.4	134.4–<175.2	175.2–<249.9	249.9–347.3
Overall TEAEs												
Grade ≤ 2, %	19 (29.2)	12 (19.4)	1 (6.7)	5 (31.3)	3 (20.0)	3 (18.8)	19 (29.2)	12 (19.4)	1 (6.7)	5 (31.3)	1 (6.7)	5 (31.3)
Grade > 2, %	45 (69.2)	49 (79.0)	13 (86.7)	11 (68.8)	12 (80.0)	13 (81.3)	45 (69.2)	49 (79.0)	13 (86.7)	11 (68.8)	14 (93.3)	11 (68.8)
Grade ≥ 4, %	20 (30.8)	25 (40.3)	7 (46.7)	5 (31.3)	7 (46.7)	6 (37.5)	20 (30.8)	25 (40.3)	6 (40.0)	8 (50.0)	6 (40.0)	5 (31.3)
Neutropenia												
Grade ≤ 2, %*	3 (4.6)	3 (4.8)	0	2 (12.5)	1 (6.7)	0	3 (4.6)	3 (4.8)	0	2 (12.5)	1 (6.7)	0
Grade > 2, %*	22 (33.8)	35 (56.5)	9 (60.0)	6 (37.5)	11 (73.3)	9 (56.3)	22 (33.8)	35 (56.5)	9 (60.0)	8 (50.0)	11 (73.3)	7 (43.8)
Grade ≥ 4, %*	17 (26.2)	23 (37.1)	6 (40.0)	5 (31.3)	6 (40.0)	6 (37.5)	17 (26.2)	23 (37.1)	5 (33.3)	7 (43.8)	6 (40.0)	5 (31.3)
Mucositis												
Grade ≤ 2, %*	20 (30.8)	32 (51.6)	8 (53.3)	6 (37.5)	9 (60.0)	9 (56.3)	20 (30.8)	32 (51.6)	6 (40.0)	8 (50.0)	8 (53.3)	10 (62.5)
Grade > 2, %*	3 (4.6)	2 (3.2)	0	2 (12.5)	0	0	3 (4.6)	2 (3.2)	0	2 (12.5)	0	0
Grade ≥ 4, %*	0	0	0	0	0	0	0	0	0	0	0	0

*Dox*doxorubicin, *Q* quartile, *C*_*min1*_ trough serum concentration at the end of Cycle 1, *C*_*avg*_ average concentration over patient’s entire treatment, *TEAEs* treatment-emergent adverse events

## Discussion

The objective of this analysis was to characterise the exposure–response relationship of olaratumab for survival outcomes and safety when combined with doxorubicin in patients with advanced STS. The combined exposure–response information for efficacy and safety was then used to optimise the dosing strategy of olaratumab and better target its therapeutic window in the ongoing confirmatory phase 3 study (NCT02451943) after accelerated approval by Food and Drug Administration and the conditional approval by the European Medicines Agency.

The exposure–response relationship of olaratumab was first characterised for survival outcomes. OS in the study was best described by a model with a constant baseline hazard, and a sigmoidal relationship for the effect of olaratumab. The C_min1_-based model yielded an EC_min1_50 estimate (66 µg/mL) corresponding to the 25th percentile of the C_min1_ distribution in the study, and a Hill coefficient indicative of a steep exposure–response relationship. The E_MAX_ estimate corresponded to a maximum decrease in the HR of approximately 75%, and was reached within the range of olaratumab serum concentration achieved in the study. Importantly, a similar exposure–response relationship was identified with the C_avg_-based model: the baseline hazard, E_MAX_, and Hill coefficient estimates were similar to those obtained in the C_min1_-based model, and the EC_avg_50 estimate (134 µg/mL) also corresponded to the 25th percentile of the C_avg_ distribution in the study. Both PK variables therefore seem to be similarly predictive of the effect of olaratumab on OS.

These findings indicate that a small increase in *C*_min1_ or *C*_avg_ in the vicinity of the EC50 is expected to lead to a dramatic change from low to near maximal OS benefit. Since the EC50 estimates correspond to the 25th percentile of olaratumab exposure in the study population, the dose of 15 mg/kg, administered on Days 1 and 8 of a 21-day cycle, therefore provides the majority of the study population with near maximum OS benefit. This is consistent with results from the PopPK analysis, where the linear clearance used to describe the disposition of olaratumab suggested that the dose of 15 mg/kg achieves serum levels leading to full target saturation [16]. This is also in line with the results of a previously published matched-case control (MCC) analysis on the same data [14] which indicated that: (1) patients in the upper three C_min1_ and C_avg_ quartiles showed an improvement in OS; (2) there was no consistent difference in OS benefit across the upper three C_min1_ and C_avg_ quartiles; and (3) HR values observed in the upper quartiles were in line with the model-predicted E_MAX_. It should also be pointed out that the ECOG PS and the number of prior lines of treatment were found to have a significant influence on OS, which is in line with the current understanding of clinical prognostic factors in STS and further supports the validity of our findings.

PFS in the study was also best described by a model with a constant baseline hazard and a sigmoidal relationship for the effect of olaratumab. The EC_min1_50 and EC_avg_50 estimates corresponded, respectively, to the median *C*_min1_ and *C*_avg_ in the study population, and the predicted E_MAX_ was lower than that for OS. In addition, the HR for PFS was not predicted to improve until the *C*_min1_ or *C*_avg_ reaches values corresponding to the 25th percentile of their distribution in the study. These findings are line with the lower activity on PFS compared with OS previously reported for olaratumab and with the previous MCC analysis on PFS which suggested that patients in the lowest exposure quartile tend to experience disease progression within the first two to three cycles of treatment.

Patients who received olaratumab in combination with doxorubicin did experience an increase in the rate of TEAEs when compared to doxorubicin alone, consistent with the toxicity profile of doxorubicin. There was, however, no apparent additional increase in the rate of TEAEs with increasing olaratumab serum levels, regardless of the TEAEs examined in the patients examined thus far. The exposure–response relationship of olaratumab for toxicity is thus very shallow, so that an increase in clinical benefit may be achieved without an increase in serious (high grade) TEAEs. The findings from the safety assessment should be interpreted with caution due to the limited number of patients that experienced TEAEs. Safety data from the ongoing confirmatory phase 3 study will provide more conclusive results.

Altogether, our analysis indicates that olaratumab has a wide therapeutic window, characterised by a steep exposure–response relationship for efficacy and a shallow exposure–response relationship for toxicity. It also indicates that the therapeutic window of olaratumab was effectively targeted by the dose of 15 mg/kg tested in the randomised phase 2 study where approximately 75% of the population were exposed to olaratumab serum levels associated with OS benefit and maximum OS benefit was potentially reached. Finally, our analysis suggests that an olaratumab *C*_min1_ of 66 µg/mL or *C*_avg_ of 134 µg/mL may represent a minimum threshold for delaying disease progression and providing OS benefit in STS.

This hypothesis was used to further optimise the dosing strategy for the ongoing randomised phase 3 study of olaratumab combined with doxorubicin (ANNOUNCE). Simulations using the PopPK model previously developed for olaratumab indicate that the use of a loading dose of 20 mg/kg on Days 1 and 8 of Cycle 1 would achieve olaratumab serum levels comparable to those observed at steady state with 15 mg/kg. The dosing strategy for the randomised phase 3 study therefore consists of a loading dose of 20 mg/kg of olaratumab during Cycle 1 followed by 15 mg/kg in ensuing cycles. This dosing strategy is expected to better target the therapeutic window of olaratumab by (1) minimising the number of patients whose C_min1_ falls below 66 µg/mL at the start of treatment; (2) replicating olaratumab steady-state serum levels associated with OS benefit; and (3) preserving the positive benefit–risk ratio of olaratumab by maintaining olaratumab serum levels with the same total range as in the randomised phase 2 olaratumab trial.

## Conclusions

The exposure–response relationship of olaratumab for PFS and OS are best described by time-to-event models with exponential hazard functions, and the effects of olaratumab on PFS and OS were well-characterized by inhibitory E_MAX_ functions with Hill coefficients. Both PK endpoints, *C*_min1_ and *C*_avg_, were equally predictive of the effect of olaratumab on OS and PFS. The model estimated maximum OS benefit was achieved by 75% of the patients in the trial, whereas only 50% of the patients are estimated to have achieved maximum benefit in PFS. The therapeutic window of olaratumab appears wide as increasing olaratumab serum concentration was not associated with increased incidence rate of TEAEs. Based on the analysis presented here and the evidence previously reported on this clinical study, a loading cycle of 20 mg/kg of olaratumab was incorporated into the confirmatory phase 3 study with the aim to prevent early disease progression and increase the number of patients that could potentially experience OS benefit.
